# Acceptability of conditions in a community-led cash transfer programme for orphaned and vulnerable children in Zimbabwe

**DOI:** 10.1093/heapol/czt060

**Published:** 2013-09-07

**Authors:** Morten Skovdal, Laura Robertson, Phyllis Mushati, Lovemore Dumba, Lorraine Sherr, Constance Nyamukapa, Simon Gregson

**Affiliations:** ^1^Institute of Social Psychology, London School of Economics and Political Science, Houghton Street, WC2A 2AE, London, UK, ^2^Save the Children, 1 St John’s Lane, EC1M 4AR, London, UK, ^3^Department of Infectious Disease Epidemiology, School of Public Health, Imperial College London, Praed Street, W2 1NY, London, UK, ^4^Biomedical Research and Training Institute, No. 10 Seagrave Road, Avondale, Harare, Zimbabwe, ^5^Catholic Relief Services, 95 Park Lane, Harare, Zimbabwe and ^6^Department of Infection and Population Health, Royal Free Hospital, Rowland Hill Street, NW3 2PF, University College London, London, UK

**Keywords:** Cash transfers, conditions, community acceptability, orphaned children, social protection

## Abstract

Evidence suggests that a regular and reliable transfer of cash to households with orphaned and vulnerable children has a strong and positive effect on child outcomes. However, conditional cash transfers are considered by some as particularly intrusive and the question on whether or not to apply conditions to cash transfers is an issue of controversy. Contributing to policy debates on the appropriateness of conditions, this article sets out to investigate the overall buy-in of conditions by different stakeholders and to identify pathways that contribute to an acceptability of conditions. The article draws on data from a cluster-randomized trial of a community-led cash transfer programme in Manicaland, eastern Zimbabwe. An endpoint survey distributed to 5167 households assessed community members’ acceptance of conditions and 35 in-depth interviews and 3 focus groups with a total of 58 adults and 4 youth examined local perceptions of conditions. The study found a significant and widespread acceptance of conditions primarily because they were seen as fair and a proxy for good parenting or guardianship. In a socio-economic context where child grants are not considered a citizen entitlement, community members and cash transfer recipients valued the conditions associated with these grants. The community members interpreted the fulfilment of the conditions as a proxy for achievement and merit, enabling them to participate rather than sit back as passive recipients of aid. Although conditions have a paternalistic undertone and engender the sceptics’ view of conditions being pernicious and even abominable, it is important to recognize that community members, when given the opportunity to participate in programme design and implementation, can take advantage of conditions and appropriate them in a way that helps them manage change and overcome the social divisiveness or conflict that otherwise may arise when some people are identified to benefit and others not.

**KEY MESSAGES**

- Soft conditions in a community-led cash transfer programme can be socially accepted.
- Soft conditions can stimulate more active community participation and help overcome social divisiveness.
- Through participation community members can mould and shape cash transfer programmes in ways that benefit them and manage change.

## Introduction

Cash transfers are emerging as key interventions for the social protection of vulnerable children in the global South. Rigorous evaluations of conditional cash transfer (CCT) and unconditional cash transfer (UCT) programmes demonstrate that both approaches, through the provision of a regular and reliable income to vulnerable households, can improve the health and developmental outcomes of children (Handa and Davis 2006; Adato and Bassett 2009; Lagarde *et al.* 2009; Arnold *et al.* 2011). Yet, the question on whether or not to condition a cash transfer remains largely unanswered and hotly debated, often guided by the underlying values of donors, government staff, policy actors and academics (Freeland 2007; Schüring 2010). In this article, we seek to contribute to the policy debate on the appropriateness of conditions by bringing to the fore perspectives of cash transfer recipients and other local stakeholders. More specifically, and in our interest to examine how local communities appropriate development interventions to fit their social landscape, we examine pathways to community acceptability of conditions in a cash transfer trial in Manicaland, Zimbabwe.

CCTs were first rolled out in Latin America where countries in the mid-1990s started to experiment with conditional welfare in response to a region-wide slowdown in economic activity and growing poverty. As suggested by the name, CCTs involve the transfer of cash to individuals or households who show some level of behavioural compliance, such as the uptake of health and educational services. The goal of CCTs is to increase the demand for government services that invest in the human capital of future generations. CCTs are therefore favoured in regions where government services are in good supply and where individuals are either (1) unaware of the benefits of using these services; (2) need the bargaining power to change behaviours or (3) presented with opportunity costs that make these services unobtainable. A few recent cash transfer trials in sub-Saharan Africa have found CCTs to outperform UCTs, particularly in relation to improving the school attendance and performance of marginalized children (Baird *et al.* 2011; Akresh *et al.* 2013). In Mexico, Barber and Gertler (2010) found conditions to have the potential to empower beneficiaries, women in particular, by enabling them, through access to information and resources, to demand their right for health care. Conditions also help cement and increase performance indicators, making conditions attractive to donors, policy makers and politicians.

Critics of cash transfer programmes have raised concerns about the social divisiveness, conflict and jealousy that may arise when some households are targeted and not others, even though they are considered poor and deserving locally (MacAuslan and Riemenschneider 2011; Ellis 2012). With regard to conditions, three perspectives on their inappropriateness dominate the field. First, and pertaining to the impact of conditions on social relations, Saucedo Delgado (2013) found, in the context of rural Mexico, that conditions contributed to a gendering of household interactions with government services—adding an extra burden to women. Some commentators warn that the failure of CCTs to redress gender roles and transfer power to recipients may result in CCTs perpetuating social inequalities (Forde *et al.* 2011). Second, concerns have also been raised about the symbolism of conditions. Nicholas Freeland (2007) argues that the term ‘conditional’ is imprecise and a perpetuation of the paternalistic mindset that took shape following the structural adjustment phase. He goes on to argue that conditionalities project know-how from the state and fail to consider the value of a partnership between grant providers and recipients. Freeland (2007, p. 77) believes that conditions send a conflicting message: ‘on the one hand, [a government] proudly tell its citizens that social protection is their basic “human right”; and then, on the other hand, threaten to deprive the neediest among them of that very “right” if they fail to meet certain “conditions” ’. Relatedly, stakeholders may find CCTs unfair, and even counterproductive, if sanctions are enforced to the most vulnerable. Yet, a lack of sanctions renders the whole concept of conditions meaningless. This suggests a need for conditions to act as guidance, best practice or achievement aims (i.e. positive motivation) rather than barriers, or a manifestation of failure (with punishment). Third, conditions increase the demand for services, yet in many parts of sub-Saharan Africa, health and educational services are facing significant constraints, which, combined with the added costs and complexities in monitoring compliance of conditions, has made some commentators question the usefulness of conditionalities in sub-Saharan African cash transfer schemes (Devereux *et al.* 2005; Schubert and Slater 2006).

These critiques and challenges highlight some very real concerns about conditions. In response, we set out to investigate the comparative effectiveness and appropriateness of CCT and UCT through a community randomized controlled trial in Manicaland, Zimbabwe. In terms of effectiveness, we found that both approaches increased school attendance among orphaned and vulnerable children and that none of the approaches managed to increase vaccination uptake (Robertson *et al*. 2013). The lack of impact on vaccination uptake can possibly be explained—although this still needs to be investigated—by a ceiling effect (i.e. uptake was close to 100%) as most children either received vaccinations at birth or through mobile distribution units, which were unaffected by the transfers. Conditions did, however, increase the percentage of children with birth registration, suggesting that conditions were somewhat more effective (Robertson *et al.* 2013). Contributing to debates on the appropriateness of conditions, the aims of this article are 2-fold. First, to investigate the overall buy-in among the general community of conditions and, second, to contextualize these findings by elaborating on local perspectives of conditions, identifying perceptions and pathways that contribute to a community acceptability of conditions.

## Methods

We draw on quantitative and qualitative data from a cluster-randomized trial of a community-led cash transfer programme in Manicaland, eastern Zimbabwe. The intervention was funded by the Programme of Support^1^ for the Zimbabwe National Action Plan for Orphaned and Vulnerable Children (UNICEF Zimbabwe), the World Bank (through the Partnership for Child Development) and the Wellcome Trust. It was implemented in partnership between the Biomedical Research and Training Institute (BRTI), the Catholic Relief Services in Zimbabwe and the Diocese of Mutare Community Care Programme (DOMCCP).

### Study location and the cash transfer programme

Manicaland is poor and most residents make a living through subsistence farming. Prior to the economic and political instability, Zimbabwe had one of the highest literacy rates in Africa, particularly among women, enhancing their orientation towards participating in development programmes (Egbo 2000). Although HIV incidence has been declining (Gregson *et al*. 2010; Halperin *et al*. 2011), HIV and AIDS continue to be a problem, particularly for children who resume caring responsibilities or are left orphaned (Campbell *et al.* 2010; Nyamukapa *et al*. 2010). To respond to the needs of the many orphaned and vulnerable children in Manicaland, a feasibility study was conducted to investigate the desirability of a cash transfer programme (cf. DD and CRS 2007). This included meetings with community members to discuss whether they would like conditions or not. Those consulted generally favoured conditions. Rather than imposing a set of conditions, the nature of the conditions included in the CCT arm were derived from initial consultations with a group of community members and other stakeholders. The conditions were obtaining birth certificates, keeping children up-to-date with vaccinations and attendance at a growth-monitoring clinic twice a year, keeping school attendance above 90% of days each month and attending parenting-skills classes.

Informed by the feasibility study, the cash transfer programme was designed to promote community engagement, with community-based cash transfer committees (CTCs) playing an active role in administering the programme (including community mobilization, beneficiary selection process, cash distributions, verification of compliance with conditions). To establish the CTCs, each community was divided into five areas, or villages, and the person from each village getting most votes was elected to become a member of the local community committee. The cash transfer programme commenced implementation in July 2009 across 30 communities.

Households eligible for cash transfers were identified through a two-stage process designed to involve community members and also to avoid bias in the selection process (see also Robertson *et al*. 2012). In the first stage, household census data, verified through a community consultation exercise, were used to establish which households in each study community met the eligibility criteria. In the second stage, communities (and, thus, their constituent eligible households) were allocated to either a control, CCT or UCT group through a random process conducted at public meetings. Through this process, a total of 2844 eligible households were allocated to receive cash transfers and a further 1199 households to the control arm of the study. The selected eligible households received bi-monthly grants of US$18 plus an extra US$4 per child living in the household (up to a maximum of three children).

To help the CCT recipients meet the conditions they were issued with ‘compliance’ cards. Service providers signed these cards during service access. They were also brought to the pay points, along with other documents such as birth certificates, child health cards and receipts for the payment of school fees. The cards and documents were checked before cash was disbursed. If a household provided a good reason for failing to meet one or more conditions, this reason was verified by the CTC. If, after a 6-month grace period with no repercussions for non-compliance, the household still did not comply with the conditions, support from DOMCCP was offered and the household was assigned a compliance buddy (local volunteer) to help meet the conditions. If after another 4 months the household remained in default, the policy was to reduce the transfer by 10%, and, after 6 months of default, the compliance buddy assumed control of the household’s transfers. Once it met the conditions, the household would receive its full transfer and the compliance buddy would be withdrawn. Reflecting the softness of the conditions, the short follow-up period over which compliance with conditions were assessed, and the adequate support provided by compliance buddies, no recipients had their funds withheld during the time of the trial because of non-compliance.

### Quantitative data

Trial endpoints were evaluated using data from a follow-up survey administered in May 2011 to eligible beneficiary households in all areas, including those in the control areas. The survey was also administered to a smaller sample (*n* = 1124) of non-eligible households. To assess local acceptability of the transfers, the survey asked household representatives whether they agreed with the following statements: ‘In the cash transfer programme, there were too many conditions’ and ‘In the cash transfer programme, the conditions were appropriate’. We present these data for eligible and non-eligible households across all three arms of the trial.

### Qualitative data

To contextualize the survey data, a qualitative study was conducted, drawing on the perspectives of 58 adults and 4 youth (age 14–21). The study participants contributed to 35 structured interviews and 3 focus group discussions. A mix of stakeholders was selected randomly from a stratified list of programme stakeholders and recruited by Shona-speaking researchers from the BRTI in consultation with community guides. As detailed in Table 1, the study participants included 24 key informants, local government employees, CTC and DOMCCP members, selected for their involvement in implementing the programme, 24 direct beneficiaries of the CCT (5 adults and 3 youths) and UCT (15 adults and 1 youth) arms as well as 14 non-beneficiaries. The sample size was determined partly by progress towards saturation, reached approximately after five interviews per adult interviewee group, and partly by the amount of time and resources available to conduct the interviews. The latter meant that we were only able to interview four youths.

**Table 1:** Summary of study participants

	Individual interviews		Focus groups	Total number of interviews
	Adults	Youth	Adults	
**Key informants**	15	0	1 (9 people)	16
**Cash transfer beneficiaries**	6	1	1 (9 people)	8
**CCT beneficiaries**	5	3	0	8
**Non-beneficiaries**	5	0	1 (9 people)	6
**Total number of people**	31	4	3 (27 people)	38
				62

Interviews (with the exception of one English language interview) were conducted in the local *Shona* language by experienced qualitative researchers. The individual interviews lasted an average of 40 min, whereas the group interviews took an average of 94 min. The topic guide used covered themes such as local understandings of the programme, cash spending, conditions, changes the programme has instigated, impact and local barriers to programme success. Interviews were translated and transcribed into English and imported into a qualitative software package (Atlas.Ti) for coding and more in-depth examination. A total of 90 codes were generated from coding the transcripts. As this article reports on the topic of cash transfers conditions, we only report on the 13 codes, or basic themes, that shed light on our quantitative findings. As illustrated by Table 2, these basic themes were arranged thematically (Attride-Stirling 2001), involving a grouping together of themes into higher order and more interpretative organizing and global themes. This process, and by analysing all the transcripts together, enabled us to move beyond description of individuals’ accounts and their individualized personal experiences of the programme (vis-à-vis their context), and instead map out some of the more prevalent experiences and perceptions as reported by the informants. To illustrate their prevalence, we have in Table 2 included a tally (percentage) of how many interviews discussed a particular theme. We will systematically discuss the basic and organizing themes emerging from this analysis.

**Table 2:** Thematic network: local perceptions of conditions

Basic theme (percentage of interviews discussing a theme)	Organizing themes	Global themes
Conditions avoid misuse of money (24)	Ensure good use of money	Conditions believed to encourage ‘good’ behaviours

Conditions ensure children benefit (21)		

Conditions help people set priorities (26)	Conditions facilitate learning and behaviour change	

Conditions sensitize people to the needs of children (26)		

Conditions encourage behaviour change (10)

Some people fail to comply to conditions (21)	People recognize there are limitations to conditions

People with conditions have little spending freedom (10)	

Poor household dynamics and situations can take priority (29)

Money spent inappropriately if there are no conditions (24)	There is a perception of a need to monitor use of money	Conditions facilitate social accountability

Difficult to monitor cash transfers if no conditions (14)		

There are consequences for those who fail to comply (16)	Conditions facilitate a sense of ownership, social responsibility and participation	

Getting ‘free’ cash comes with a responsibility (21)	

Community members take an active role in monitoring compliance (26)

Ethical approval for this study was granted by the Imperial College Research Ethics Committee (ICREC_9_3_10), the BRTI’s Institutional Review Board (AP81/09) and the Medical Research Council of Zimbabwe (MRCZ/A/1518). Informed and written consent was gathered from all participants with the agreement that their identities would not be revealed. Pseudonyms have therefore been used throughout.

## Results

The findings of this study must be read against the background that this cash transfer programme was community-led and directed. Its design, including the ‘soft’ conditions, was developed through community consultations and a feasibility study. Community-elected CTC members took an active role in administering the programme such as playing a key role in the selection of beneficiaries and in monitoring compliance. Elsewhere, we have highlighted the importance of integrating cash transfer programmes into a community context to achieve acceptability of cash transfers and maximize impact (Skovdal *et al*. 2013). It is against this context we now present our findings.

### Community acceptability of conditions

In total, 5167 households were interviewed at follow-up. Of these, 4043 were eligible at baseline for the CT programme and 1124 were not eligible. Table 3 shows a general and widespread acceptance of conditions in our programme areas. In fact, eligible households in the CCT areas were the most likely to think that the conditions were appropriate (92.2% compared with 74.6 and 70.9% among eligible households in the UCT and control areas, respectively, chi-squared test *P* < 0.001). However, the eligible households in the CCT areas were also somewhat more likely to think there were too many conditions (7.6% of eligible CCT households thought this compared with 5.1 and 3.6% among eligible UCT and control households, respectively, chi-squared test *P* < 0.001).

**Table 3:** Community acceptability of conditions by household eligibility status and trial arm

		Eligible households			Non-eligible households		
		Control	UCT	CCT	Control	UCT	CCT
The conditions were appropriate	Agree	70.9%	74.6%	92.2%	82.8%	69.4%	83.5%
	Disagree	7.9%	13.2%	5.4%	9.2%	10.0%	11.1%
	Don’t know	21.3%	12.2%	2.4%	8.0%	20.6%	5.4%
	*N*	1115	1451	1247	436	389	297

There were too many conditions	Agree	3.6%	5.1%	7.6%	5.3%	3.3%	3.4%
	Disagree	74.0%	81.5%	89.1%	86.2%	74.6%	90.2%
	Don’t know	22.4%	13.4%	3.3%	8.5%	22.1%	6.4%
	*N*	1116	1450	1248	436	389	297

Among eligible households, there were no significant differences between perceptions of the conditions by sex of respondent—men and women were equally likely to perceive the conditions as appropriate and to agree that there were too many conditions (Table 4).

**Table 4:** Gender differences in community acceptability

		Control arm		UCT arm		CCT arm	
		Female respondents	Male respondents	Female respondents	Male respondents	Female respondents	Male respondents
The conditions were appropriate	Agree	70.0%	68.8%	75.6%	73.1%	93.0%	90.5%
	Disagree	8.1%	6.3%	12.2%	16.6%	4.9%	6.3%
	Don’t know	21.9%	24.9%	12.3%	10.3%	2.0%	3.2%
	*N*	807	205	1052	271	891	221

There were too many conditions	Agree	3.5%	4.9%	4.9%	4.8%	7.3%	9.5%
	Disagree	74.0%	67.8%	82.0%	83.8%	89.9%	86.9%
	Don’t know	22.5%	27.3%	13.1%	11.4%	2.8%	3.6%
	*N*	808	205	1051	271	891	222

### Local perceptions of conditions

Shedding light on this overwhelming acceptability of conditions, informants spoke of how conditions entice behaviours that are locally embraced and facilitate social accountability of ‘free’ money (see Table 2).

#### Conditions believed to encourage ‘good’ behaviours

Interviewees and focus group participants often spoke about the potential of conditions to entice and facilitate more socially desirable behaviours that ensure good use of money and ultimately benefit vulnerable children. Cementing this perspective was a commonly held view that conditions help circumvent a misuse of money.

I think the conditions were good, receiving money without any conditions doesn’t work because you won’t pay school fees or do anything constructive with the money. It is better to have conditions. (Anashe, female, caregiver benefiting from CCT)

This view was held by all kinds of stakeholders, including those benefiting from CCTs and UCTs, despite the fact that even the unconditional arm increased educational attendance (Robertson *et al*. 2013). It was therefore not an expression of stigma or jealousy, but rooted in a common recognition of what good parenting is and a common goal to support children. For example, to children and youth benefiting from the cash transfers, conditions presented them with a reassurance that they were to benefit from the programme and that the money would not be spent frivolously by their parents.

The conditions are good, because had it been that money is just given without them, parents are the ones who would be benefiting and not children. They will be using the money on what they want only. So if they are told the money has conditions it will be clear to them that the money is not for them alone. (Dzingai, 15-year-old boy benefiting from CCT)

Also alluded to by Dzingai, is the notion that conditions can strengthen the bargaining power of household members whose interests are closely aligned with the objectives of the programme and the implementing agency. For Dzingai, conditions helped ensure that the interests of the child were prioritized. Similarly, Kuasa, a CTC member, spoke about how conditions may reduce the likelihood of men using the money to their own benefit, and providing women, children and community members with the opportunity to question their use of the money.

I recommend money with conditions than without conditions because people will be compelled to improve the livelihoods of their households unlike money without conditions where people will just use the money for whatever they want. There are some men who can take the money and go and marry a second wife but his children will be suffering in the household just because the money did not come with conditions. No one will dare to ask him what he did with the money if there are no conditions. (Kuasa, female, CTC member)

In addition to sending a message about how best to spend the money, conditions were said to facilitate learning and encourage behaviour change. A number of informants, for example, spoke about how conditions encouraged recipients to think about what is important for children and to prioritize their needs.

There is a big difference between the two groups because the group that was receiving money with conditions got a lot of knowledge unlike the other group. Like if you take your child for vaccination, then your child will have good health. If your children have birth certificates, then they have a good life ahead of them. If you have a national identity card, you are a free citizen in your country. The households without conditions just spend the money they were receiving on anything they wanted without anyone asking any questions. Some households did not even send their children to school. It’s called for a wise parent to send their children to school. (Edzai, female non-benefiting community member)

People got informed on the importance of caring for the children … it is good because it makes people think about their children. (Ruko, male, CTC member)

The idea that conditions help parents become ‘wise’ was frequently mentioned in the interviews. This was particularly the case when conditions helped change the attitudes and behaviours of parents who were believed to engage in poor parenting practices. One such example is the perceived changing attitude of people within the apostolic faith who, in this context and for religious reasons, have a poor record of taking their children to hospital. Although some increases in uptake of multi-dose vaccines not given at birth were observed, these were not statistically significant (Robertson *et al*. 2013). It was however believed that Apostolics in particular benefited from the programme.

We are noticing an improvement on the issue of the Apostolics and their use of hospital services. Some are responding positively and at least they now know that children are supposed to go to the hospital. (Rindi, male, local government employee)

Not all families were believed to successfully comply with the conditions, primarily because of the self-interest of a few individuals. Raviro, a CTC member, was of the impression that a few men in the community used the money to go drinking, neglecting the needs of children in their household.

There are some men in the community who had difficulty in understanding the programme. These men do not work, they just stay at home. So when the money came, they took the money and used it to meet their own needs instead of using it for the benefit of their children. Instead of paying for school fees and buying uniforms, the men went drinking or paid off their own debts. (Raviro, male, CTC member)

For other benefiting households, their sheer deprivation (e.g. lack of food) meant they struggled to prioritize their children’s schooling and thus meeting the conditions.

While conditions were described as playing an important role in guiding parents to make decisions that improve the health and development outcomes of their children, it is difficult to attribute improvements in health and development outcomes to conditionalities alone. Rather than being the conditions themselves that bring about this change, it could be a combination of increased awareness and parents’ natural inclination to do the best for their children. For example, one UCT recipient said: ‘I received money without conditions […] when I received the money I went straight to pay school fees for my children. I didn’t want them to miss school’. Indeed, our effectiveness study found only small differences in the effects of CCT and UCT programmes on the school attendance of vulnerable children (Robertson *et al*. 2013). Without undermining the importance of conditions in facilitating learning and a focus on children’s needs, it is arguably the perception of the added benefits of conditions as outlined in this section, as well as the counselling and support that went with, what, in practice, were ‘soft’ conditions, that contributed to this overwhelming buy-in and appreciation of conditions.

#### Conditions facilitate social accountability

Another pathway to community acceptability of conditions pertains to the opportunities for social accountability that conditions present. There was, for example, a general belief, deepened by the perception that UCTs are more likely to be misappropriated, that the money distributed needs to be monitored, and that conditions make this more feasible.

The difference is that, if a person is getting cash without conditions, the person can use the money to do whatever they want or even misuse the money because they will say there is not going to be a follow-up. (Pepukayi, 21-year-old male household head benefiting from CCT)

The best way forward is to have conditions. They prevent people from doing the wrong things because, where there are conditions, they help in monitoring. If there were no conditions, you wouldn’t be monitoring anything and there would be no way of knowing if the programme was really working. (Shamu, male, representative from implementing agency)

The fact that CCT recipients, through a monitoring system, were held accountable for the money they received, appeared to make it easier for non-beneficiaries to justify and accept that some community members got money. Conditions, in the context of this community-led cash transfer initiative, provided community members with the opportunity to participate in the monitoring of the programme.

People would say we don’t want to see people buying snacks […]. That money should be spent effectively. People would just say this in passing to those people who would have received the money. This was not said with harsh words but it was said in a nice way to encourage people to be more responsible. You say it nicely in a social way so that they understand. (Zira, male, non-benefiting community member)

Although such social control and regulation can be problematic, no cash transfer beneficiaries reported being hassled by community members, suggesting that community members, like Zira, articulated their support and encouragement in a sensitive way.

Some cash transfer recipients were surprised by the opportunity to be given ‘free’ money and felt that one way to give back, or work for the money, was to respect the programme aims and spend the money accordingly.

What surprised me is that people give you money to use which you didn’t work for. I said to myself “I will use the money appropriately.” (Tinashe, female, caregiver benefiting from UCT)

They effectively took conditions, or the social control, as an opportunity to actively participate in the programme. Getting ‘free’ money comes with a responsibility to use the money as intended, a recognition most cash transfer recipients endorsed.

It’s a good thing to have conditions. If people are getting money without working for it, they should use the money properly. The advantage is that people know the use of the money they are getting, like paying school fees for their children and sending their children to clinic. So far I have not seen any disadvantages of the conditions. (Pepukayi, 21-year-old male household head benefiting from CCT)

Although some cash transfer recipients complained about getting insufficient funds to meet the conditions (the spending freedom of recipients was dependent on the cost of their children’s education and the availability of household assets), others, like Anashe who had a little spare money, commented on how the programme enabled decision making.

If you have conditions you are given guidelines, you use the money appropriately […] if you use the money appropriately then you can use the rest in your own way and relax. (Anashe, female, caregiver benefiting from CCT)

Many cash transfer recipients who did find themselves with some spare money joined savings groups with the intention of investing in household assets, such as buying livestock or poultry. Through such activities, advice and encouragement, recipients took an active role in ensuring programme goals were met, projecting themselves as active participants in the programme as opposed to passive recipients of aid.

We discuss issues to do with cash transfers and how we are using this money, advising each other to use the money properly and not abuse the money but to use it on our children. (Gamba, male, caregiver benefiting from UCT)

## Discussion

The findings presented in this article point to a widespread acceptance of conditions in the context of a community-led cash transfer initiative. We identified two key pathways leading to this acceptance. One, conditions were viewed positively and seen as reasonable, a proxy for good parenting and guardianship. Two, social recognition of the need to be held accountable for the ‘free’ money provided recipients and community members with an action pathway as contributing players rather than passive recipients. In effect, the conditions made explicit a social contract between the beneficiaries, their community and the implementing agencies—recognizing all stakeholders, including recipients, as actors in the programme. This not only improved the bargaining power of women and children, enabling households to prioritize the needs of children, it may also have helped recipient households overcome the stigma attached to welfare payments.

Our experiences suggest that conditions can, if designed by and implemented through local community structures, be beneficial and overcome the social divisiveness or conflict that may arise when some people are identified to benefit and others not. In fact, our experiences suggest that conditions, in an oddly contradictory way to the meaning of the word, provided community members with an incentive and opportunity to actively engage with the programme—empowering them as actors. Furthermore, our sanctions were enabling and did not, in fact, remove the cash transfer, but diverted it to a responsible buddy, altering control and not receipt. If the conditions themselves were not viewed positively, for example, if people felt the programme was imposing ‘hard’ and unfair sanctions, we might have witnessed a different outcome.

As it is the most vulnerable and hard-to-reach children who will suffer if parents fail to meet the conditions, there is a need for a discussion on the kind of sanctions that one would impose on those who fail to meet the conditions as well as the meaning of conditions. As detailed earlier in the article, our conditions were ‘soft’ and agreed upon by a group of community members at the design stage. For our informants, the conditions cemented a social contract between all actors, contributing to a sense of accountability. As such, and reflecting Scott and Marshall’s (2005) definition of social sanction, we found that the conditions did not have to be ‘hard’ and enforced to have an impact. The anticipation of being socially recognized as a good parent (reward) or the off-chance of being withdrawn from the programme (punishment), meant that people conformed to the conditions. Although people’s perceptions of a cash transfer programme may change if, and when, they realize that the conditions are not enforced or if the organization enforces conditions in a hard-lined manner, it is the social control of fair conditions, sparked by community involvement and a community-wide sensitization to the needs of orphaned and vulnerable children that appear to have been in force in this programme, facilitating agency, participation and buy-in.

It is also possible that the overwhelming support for conditions, fuelled by community perceptions that unconditional transfers are likely to be misappropriated, also contributed to an implicit social contract between UCT recipients and community members. Indeed, as outlined in this article, UCT beneficiaries also sensed the need to spend the money responsibly, and our effectiveness study found both CCTs and UCTs to promote educational outcomes (Robertson *et al*. 2013). Gaarder (2012) recently discussed, albeit without empirical support, the potential of social sanctions or peer monitoring, to act in a similar way as conditions, underlining the importance of exploring this potential further.

As we sought to map out the stock of symbolic resources and meanings used by our informants to make sense of conditions, the qualitative study did not pursue a more detailed analysis of individual experiences or patterns between the different types of respondents participating in the study. Another limitation of this study pertains to response bias. We recognize that some participants may have provided us with desirable responses to show their gratefulness and did not want to challenge the programme out of fear for it being withdrawn. To mitigate the risk of surveyor-induced responses we made sure the survey team (researchers from BRTI) was not involved in delivering the intervention (the responsibility of DOMCCP), and that we only used trained and experienced research assistants who were able to establish rapport and openness.

## Conclusion

Our experiences suggest that conditions can be embraced, particularly if they are considered fair, a proxy for good parenting, achievement and merit, and enable community members to take an active role in the programme and not sit back as passive recipients of aid. We conclude that cash transfer programmes can make considerable progress in overcoming some of the unintended consequences they may present, if community members participate in their design and implementation, giving them the opportunity to mould and shape cash transfer programmes in ways that benefit them and fit their social landscape.

## Endnote

^1^ The Programme of Support is funded by UKaid, Ausaid, Swedish International Development Agency, New Zealand Aid, Dutch Government and German Government.
